# Infant Nutrition and Other Early Life Risk Factors for Childhood Obesity According to Disability Status

**DOI:** 10.3390/nu15204394

**Published:** 2023-10-17

**Authors:** Melissa K. Blake, Ruixuan Ma, Erika Viana Cardenas, Parisa Varanloo, Yaray Agosto, Carolina Velasquez, Katheryn A. Espina, Joanne Palenzuela, Sarah E. Messiah, Ruby A. Natale

**Affiliations:** 1 Department of Pediatrics, University of Miami School of Medicine, Mailman Center for Child Development, 1601 NW 12th Ave, Miami, FL 33136, USA; epv11@med.miami.edu (E.V.C.); pxv177@miami.edu (P.V.); yagosto@med.miami.edu (Y.A.); cxv308@miami.edu (C.V.); kxe258@med.miami.edu (K.A.E.); jpalenzuela@miami.edu (J.P.); rnatale@med.miami.edu (R.A.N.); 2 Division of Biostatistics, Department of Public Health Science, University of Miami School of Medicine, 1120 N.W. 14th Street, Miami, FL 33136, USA; rxm1424@med.miami.edu; 3 University of Texas Health Science Center at Houston School of Public Health, 2777 North Stemmons Freeway, Suite 8400, Dallas, TX 75207, USA; sarah.e.messiah@uth.tmc.edu; 4 Center for Pediatric Population Health, University of Texas Health Science Center at Houston School of Public Health, 2777 North Stemmons Freeway, Suite 8400, Dallas, TX 75207, USA

**Keywords:** pediatric obesity, disability, preschool age, infant nutrition, birth weight, premature birth, ethnicity

## Abstract

One in five preschool-aged children in the United States is obese, and children with disabilities are significantly impacted. This study aimed to determine the association between age at solid food initiation and obesity prevalence in preschool-aged children while considering disability status, ethnicity, gestational age, and birth weight. Analysis was conducted on a sample of 145 children aged 2 to 5 years who were enrolled in ten childcare centers. Parents completed a survey assessing disability status, race and ethnicity, birth weight, gestational age, and age of solid food initiation. Height and weight were collected concurrently. Multivariable logistic regression models generated the odds of developing obesity based on age at solid food initiation, disability status, ethnicity, gestational age, and birth weight. There was no significant difference in the odds of being obese based on age at solid food introduction. Children with disabilities (OR = 0.17, 95% CI 0.04–0.6, *p* = 0.01) and children born preterm (OR = 0.28, 95% CI 0.08–0.79, *p* = 0.03) had significantly lower odds of being obese. Hispanic children (OR = 4.93, 95% CI 1.91–15.32, *p* = 0.002) and children with higher birth weights (OR = 1.47, 95% CI 1.17–1.92, *p* = 0.002) were more likely to be obese. With pediatric obesity rates continuing to rise, these findings can inform future intervention efforts.

## 1. Introduction

One in every five children in the United States is affected by obesity [1]. Childhood obesity has been linked to various chronic conditions, including type 2 diabetes, cardiovascular disease, and certain types of cancer [2]. Previous studies show that obesity in childhood tracks strongly into adulthood [3]. These findings underscore the importance of addressing childhood obesity as an early intervention strategy to mitigate the long-term health complications associated with obesity in adulthood.

One area targeted for interventions aimed at the early primary prevention of obesity is the development of early nutrition patterns during infancy. Currently, the American Academy of Pediatrics recommends delaying the introduction of solid foods until the age of six months due to the increased risk of unhealthy weight gain in infancy [4]. Previous research revealed that formula-fed infants who received solid food before four months of age had a significantly higher risk of developing obesity at the age of three years [5]. Recent literature, however, has presented mixed findings on this topic [6,7,8]. A recent systematic review examining the relationship between the timing of introducing complementary foods and the risk of obesity found no significant association between the timing of solid food introduction and the development of overweight or obesity in children [8]. 

Although the impact of early nutrition habits on the risk of developing obesity in childhood yields mixed results, there are several other factors that have consistently been associated with an increased risk. Notably, children who belong to race and ethnic minority groups have a significantly higher risk of developing childhood obesity [9,10,11,12]. These race/ethnic group disparities have been attributed, in part, to rapid weight gain during infancy [9]. Additionally, preterm birth and low birth weight have consistently been linked to higher rates of obesity and the development of other chronic conditions, such as type 2 diabetes and coronary artery disease, in adulthood [13,14,15]. 

One notable risk factor that lacks investigation in the literature is disability status, particularly at the intersection of race/ethnicity. In 2019, more than three million children in the United States were documented as having a disability, affecting over 2.6 million households [16]. Children with disabilities have a two to three times higher risk of developing overweight and obesity compared with children without disabilities [17]. While it is evident that having a disability increases the risk of developing obesity in children, the precise underlying mechanism for this association remains unclear.

Given the limited understanding of how early nutrition habits and disability status impact the risk of childhood obesity, further investigation is needed, especially among racially and ethnically diverse children. The objective of this study was to examine the relationship between the timing of solid food introduction and obesity rates among preschool-aged children, while considering factors such as disability status, ethnicity, term at birth, and birth weight. We hypothesized that introducing solid foods before six months of age would be associated with an increased risk of unhealthy weight in preschool-aged children. Additionally, it was hypothesized that having a disability, identifying as a race or ethnic minority, having a history of preterm birth, and having low birth weight would also be associated with an unhealthy weight in preschool-aged children.

## 2. Materials and Methods

### 2.1. Participants and Procedures

Participants were recruited from ten childcare centers (CCCs) in Miami-Dade County, Florida, that served children from economically disadvantaged backgrounds. Of the ten CCCs that were recruited, at least ten children were enrolled from each center, and at least 20% of recruited children had a recorded disability. Consent was obtained from parents from a larger randomized control trial designed to investigate an obesity prevention program’s impact on childhood BMI (Healthy Caregivers–Healthy Children (HC2)). Five CCCs were chosen to receive the obesity prevention program. HC2 consists of 24 lesson plans designed to improve nutrition and physical activity, both in the children and in the CCC environment. The five remaining CCCs received a mental health attention control curriculum (matched on exposure time). These toolkits provided guidance for directors and teachers on the promotion of adequate child nutrition and the management of challenging behaviors in children with disabilities.

Data collection occurred between August and December 2021, following the commitment of each CCC director to participate in the study. Research assistants engaged with parents during drop-off and pick-up times to establish contact. At this time, parents were recruited through the distribution of consent forms and baseline survey packets, which they could complete at home, and which were available in both English and Spanish. If parents faced challenges due to low literacy levels, research assistants were available to provide assistance with form completion. Once consent forms were completed, research assistants revisited each participating CCC to collect the height and weight measurements of the consented children, following the guidelines set by the Centers for Disease Control and Prevention (CDC).

To ensure consistent and unbiased implementation of the study, research assistants completed mandatory training for one week, focusing on the assigned research protocol and methods. The training was conducted by a multidisciplinary team comprising a clinical child psychologist with expertise in obesity prevention in childcare settings, a mental health counselor with a disability background, a physical therapist experienced in special needs, and a clinician specializing in special education. The training involved a combination of on-site activities and role-play scenarios designed to address potential biases related to obesity or disabilities in children. Research assistants strictly followed a step-by-step implementation protocol within the CCC. To ensure fidelity, program managers conducted an evaluation at the beginning of each study and followed up one month later. A total of 220 parents provided consent for their children, who were aged between two and five years old, to participate in the study and completed our baseline survey packets. This study received approval from the Institutional Review Board. 

### 2.2. Measures

#### 2.2.1. HC2 Caregiver Interview to Document Age at Solid Food Initiation

Age, sex, race/ethnicity, and birth weight (lbs, oz) were collected for each participant in the HC2 baseline questionnaire. Caregivers were asked if their child was born full-term or preterm (less than 37 weeks of gestational age). Caregivers were also asked to identify the age (months or years) at which their child initiated solid foods. Children were then stratified into two groups based on whether they initiated solids before or after six months of age. 

#### 2.2.2. Anthropometry 

The height (cm), measured with a stadiometer, and weight (kg), measured with a digital scale, of each child was collected and used to calculate an age- and sex-adjusted BMI percentile. Data were collected based on guidelines from the US Department of Health and Human Services [18]. Each child’s height and weight was converted to a BMI percentile that was adjusted for age and sex according to the World Health Organization (WHO) growth standard charts used to monitor the growth of children aged from birth to younger than two years, CDC 2000 growth reference charts to monitor the growth of children aged two to twenty years without obesity, and CDC 2022 extended BMI-for-age growth charts for children and adolescents aged two to twenty years with obesity [19,20,21]. The children’s BMI percentiles for age and sex were categorized into four categories: (1) underweight—with a BMI percentile for age and sex below the 5th percentile; (2) normal weight—with a BMI percentile for age and sex between the 5th and 85th percentiles; (3) overweight—with a BMI percentile for age and sex between the 85th and 95th percentiles; (4) obese—with a BMI percentile for age and sex equal to or greater than the 95th percentile. For our analysis, we defined the outcome assessment as a binary variable: unhealthy weight (BMI percentile greater than or equal to the 95th percentile) versus healthy weight (BMI percentile less than the 85th percentile).

#### 2.2.3. Disability Status and Ethnicity

Disability status and history of meeting developmental milestones were defined based on answers to the following HC2 baseline questions (Y/N): (3) Does this child have a documented disability? (4) Did a teacher, doctor, or health professional ever tell you they have a concern about this child’s learning, development, or behavior? (5) Do you have any concerns about this child’s learning, development, or behavior? (6) Are you concerned about how this child (a) talks and makes speech sounds; (b) understands what you say; (c) uses (his/her) hands and fingers to do things; (d) uses (his/her) arms and legs; (e) behaves, (f) gets along with others; (g) is learning to do things for (himself/herself); (h) is learning preschool or school skills? (7) Has this child ever been referred to Early Steps or the Florida Diagnostic and Learning Resources System (FDLRS) or Miami-Dade Public Schools for testing or evaluation? If a parent answered “yes” to question (3), their child was considered to have a confirmed disability. Answering “yes” to any part of questions (4), (5), or (6) classified a child as having a suspected disability. Answering “yes” to question (7) classified a child as having a referred disability. Participants who did not answer “yes” to any part of questions (3), (4), (5), (6), or (7) were classified in the non-disability group. Caregivers were also asked to indicate their own country of birth and whether or not their child was English proficient. 

### 2.3. Sample Size Calculation

This analysis consisted of baseline data from a larger cluster randomized controlled trial. Clusters were considered to be childcare centers as the primary unit of analysis. This is an individual-level post hoc analysis. As this is a retrospective analysis using our baseline data, we performed a post hoc power analysis and found that our study had ample (>90%) statistical power to detect differences in obesity status (>10% of the population) and disability status (>10% of the population) at the alpha level of 0.05. 

### 2.4. Statistical Analyses 

Child characteristics and early-life nutrition patterns were summarized as a mean with standard deviation for numerical variables such as participant age at enrollment and birth weight in pounds, and as a count with percentage for categorical variables such as participant gender, disability status, and ethnicity. The association between the timing of introduction of solid foods in months with participant characteristics and early-life nutrition patterns in the overall sample and in samples stratified by disability status (Non-disability versus Suspected, Referred, and Confirmed disability combined) were compared using the Wilcoxon rank–sum test, Pearson’s chi-squared test, and Fisher’s exact test, depending on the distribution of the variables. 

Multivariable logistic regression models were used to examine the association of the timing of introduction of solid foods with developing an unhealthy weight (BMI percentile ≥ 95th percentile versus BMI percentile < 85th). Potential confounders, including disability status (Non-disability, Suspected disability, Referred disability, and Confirmed disability), race/ethnicity, term at birth, and birth weight, were adjusted for in the final models. Multivariable logistic regression models were also fit for examining the association of disability status, term at birth, ethnicity, and birth weight with developing obesity, while adjusting for the potential confounders of English proficiency and family members’ countries of birth. Any *p*-value less than 0.05 was considered statistically significant. All tests were two-sided. The analysis and graphical visualization were performed using R [22].

## 3. Results

Data were collected from a total of 145 participants, and their demographics and early nutrition patterns are presented in Table 1, stratified by the timing of solid food introduction and disability status. Within the overall sample of 145 participants, 9% of participants (*n* = 13) reported initiating solid foods before 6 months of age, while 91% of participants (*n* = 132) introduced solid foods at 6 months of age or later. Of the 145 total participants, 55% of participants (*n* = 80) had a suspected, referred, or confirmed disability, while 45% of participants (*n* = 65) did not have a disability.

The majority of participants who initiated solid foods early (62%) belonged to the non-disability group. Furthermore, among those who initiated solids early, a significant proportion identified as Hispanic (69%), had limited proficiency in English (62%), or had at least one caregiver who was born overseas (75%). In the non-disability group, participants who were introduced to solids before 6 months of age had a higher average birth weight compared with participants with later solid food initiation. Regarding term at birth, the majority of participants in both the non-disability group (100%) and the disability group (80%) who initiated solid foods early were born full term.

Examining BMI, 38% of participants with early solid food initiation and 31% of participants who initiated solids on time were obese. Concerning disability status, the majority of participants without a disability who were classified as obese initiated solids early. It is worth noting that Hispanic participants were more likely to be obese regardless of the age at solid food initiation, although the majority of Hispanic participants who were obese were introduced to solid foods early. These observed differences, however, did not reach statistical significance.

In the univariable and multivariable logistic regression analyses in Table 2, the introduction of solid foods at or after six months of age was associated with lower odds of being obese compared with children who initiated solid foods early when controlling for disability status, ethnicity, birth term, and birth weight. This association, however, was not found to be statistically significant in univariable (OR = 0.72, 95%CI 0.23–2.51, *p*-value = 0.590) or multivariable (OR = 0.59, 95% CI 0.12–2.74, *p*-value = 0.510) models. When considering disability status, having a confirmed disability was associated with decreased odds of being obese between two to five years of age (OR = 0.18, 95% CI 0.05–0.52, *p*-value = 0.003). Having a suspected or referred disability was also associated with decreased odds of being obese; however, this was not statistically significant. Children who were born preterm were less likely to develop obesity compared with children born at full term (OR = 0.22, 95% CI 0.06–0.61, *p*-value = 0.010). Children with higher birth weights were more likely to be obese (OR = 1.47, 95% CI 1.17–1.92, *p*-value = 0.002). Hispanic children were also more likely to be obese compared with non-Hispanic children (OR = 4.93, 95% CI 1.91–15.32, *p*-value = 0.002).

## 4. Discussion

The timing of solid food introduction is an important topic for parents and has been strongly thought to impact childhood obesity [4,5,6,7,8]. Contrary to our primary hypothesis, this study did not find a significant association between early solid food initiation and obesity in preschool-aged children. Furthermore, contrary to studies showing an increased risk of obesity in individuals with disabilities, our study found that having a disability was protective against obesity in early childhood [17]. Consistent with other research, our results showed that young children from race and ethnic minority backgrounds were more likely to have obesity while those with low birth weights or preterm births were less likely to have obesity. Although other studies have suggested a link between the early initiation of solid food and an increased childhood obesity risk, more recent studies have shown mixed results similar to our findings here [23,24]. These findings have important implications for the current obesity epidemic and can help inform targeted obesity prevention strategies for young children. 

Due to only 9% of participants in our study initiating solid foods before six months of age, it is challenging to determine whether the lack of a significant association between early solid food initiation and obesity was due to biopsychosocial factors or simply our limited sample size. Previous studies have proposed that the early initiation of solid foods may lead to obesity via the disruption of exclusive breastfeeding, which plays a crucial role in infants learning to self-regulate their feeding [25,26]. However, as we did not collect breastfeeding data, it is difficult to ascertain the impact of this factor on our results. Additionally, our lower reported prevalence of early solid food initiation compared with national data suggests an increased adherence to pediatric nutrition guidelines, which recommend against introducing solid foods before 6 months of age [5,27]. Parental adherence to guidelines may be influenced by frequent conversations with pediatricians, which build trust and promote adherence [28].

Contrary to our hypothesis and current data, our study revealed that having a confirmed disability was associated with 83% reduced odds of developing obesity [29,30,31]. Only 6% of participants in the disability group reported an early initiation of solid foods, while the remaining 94% introduced solids after 6 months of age. Parents of children with disabilities may prioritize discussing their child’s health conditions during well-child visits, which can lead to increased adherence to pediatric guidelines and recommendations for nutrition [32]. However, it is possible that disability status impacts obesity risk later in childhood rather than during infancy and preschool, thus leading to an increased risk of obesity later in life. 

Regarding ethnicity, our study confirmed our hypothesis that identifying as Hispanic increases the risk of childhood obesity. Only 9% of Hispanic participants initiated solid foods early, which is markedly lower than the national average [27]. One potential cause for this may be the increased number of children with disabilities in our sample. The increased overall risk for developing obesity in Hispanic participants in our study may therefore be secondary to ethnic or cultural factors, given that cultural and language barriers have been shown to negatively impact outcomes, including BMI, in minority populations [33,34]. Disparities in obesity risk among Hispanic children highlight the need for interventions that address language barriers and promote culturally sensitive care [35].

Contrary to our hypothesis, our findings indicated that participants born preterm or with low birth weights had significantly reduced odds of developing obesity. It is worth noting that the majority of these participants initiated solid foods after six months of age. While the impact of birth term and weight on BMI is classically thought to be secondary to accelerated weight gain during infancy, other studies have found increased birth term and birth weight to be positively associated with obese BMIs during adolescence [14,36,37,38]. In line with these studies, our findings suggest that preterm birth and low birth weight may protect against obesity during early childhood. It is then possible that the impact of birth term and weight may manifest later in childhood or during adolescence rather than during the preschool years. 

### Study Strengths and Limitations

Strengths of this study include our ethnically diverse sample; however, due to Hispanic participants being the majority group, the findings here may not be generalizable to other race and ethnic groups. In addition, the term Hispanic constitutes many subgroups, and South Florida is home to many of resident groups from Cuba and Central and South America as well as the Dominican Republic and Puerto Rico. Generalizing the results to other subgroups should therefore be further explored. Limitations of the current study include self-reported data, which may be at risk for recall bias, given the amount of time that may have passed between the child’s birth and the current assessment. A second limitation is that the questionnaire did not include questions about breastfeeding, which has been shown to be protective against obesity in early childhood [39]. 

## 5. Conclusions

With pediatric obesity continuing to remain an important clinical and public health challenge, it is imperative that emphasis be placed on further developing primary obesity prevention strategies in young children. This is particularly true for children with disabilities as they are disproportionately impacted. While our study found that early solid food initiation does not significantly increase the risk for developing obesity during early childhood, our results do indicate a need to further evaluate the impact of disability status on childhood obesity risk. Additional efforts should be made to better understand the precise age or period during which disability status begins to impact obesity risk. Likewise, future studies should also focus on developing methods to decrease ethnic disparities in pediatric obesity, particularly in Hispanic youth.

## Figures and Tables

**Table 1:** Participant Characteristics and Nutrition Patterns by Introduction of Solids (months) and Disability Status.

	Introduction of Solids (<6 or ≥6 Months)								
	Total (*N* = 145)			Non-Disability (*N* = 65)			Suspected, Referred, and Confirmed Disability (*N* = 80)		
	<6 (*N* = 13)	≥6 (*N* = 132)	*p*	<6 (*N* = 8)	≥6 (*N* = 57)	*p*	<6 (*N* = 5)	≥6 (*N* = 75)	*p*
Child Characteristic									
Age, (yrs, Mean(SD))	3.40 (0.96)	3.10 (1.03)	0.30	3.70 (0.92)	3.30 (1.18)	0.40	2.80 (0.80)	2.94 (0.88)	0.80
Sex, *n* (%)			0.20			0.50			0.40
Female	4 (31)	64 (48)		3 (38)	30 (53)		1 (20)	34 (45)	
Male	9 (69)	68 (52)		5 (62)	27 (47)		4 (80)	41 (55)	
Birth Weight (lbs, Mean(SD))	6.67 (1.58)	6.13 (1.99)	0.50	7.29 (1.11)	6.46 (1.57)	0.16	4.50 (0.71)	5.87 (2.25)	0.20
Gestational Status, *n* (%)			0.20			0.60			0.60
Full-term	12 (92)	91 (73)		8 (100)	46 (88)		4 (80)	45 (62)	
Preterm	1 (8)	34 (27)		0 (0)	6 (12)		1 (20)	28 (38)	
Ethnicity, *n* (%)			0.50			0.90			0.90
Non-Hispanic	2 (18)	39 (31)		1 (12)	12 (22)		1 (33)	27 (38)	
Hispanic	9 (82)	87 (69)		7 (88)	43 (78)		2 (67)	44 (62)	
Country Born, *n* (%)			0.30			0.90			0.14
Not United States	9 (75)	74 (58)		5 (62)	32 (60)		4 (100)	42 (57)	
United States	3 (25)	53 (42)		3 (38)	21 (40)		0 (0)	32 (43)	
English is Proficient, *n* (%)	5 (42)	80 (64)	0.20	4 (50)	27 (50)	0.90	1 (25)	53 (75)	0.06
Weight Group, *n* (%)			0.60			0.40			0.60
BMI ≥ 95th percentile	5 (38)	41 (31)		5 (62)	24 (42)		0 (0)	17 (23)	
BMI < 85th percentile	8 (62)	91 (69)		3 (38)	33 (58)		5 (100)	58 (77)	

**Table 2:** Crude and Adjusted Logistic Regression Predicting Childhood Obesity (BMI ≥ 95th Versus <85th Percentile).

		Univariable Analysis		Multivariable Analysis	
Predictor		Odds Ratio (95% CI)	*p*-Value ^c^	Odds Ratio (95% CI)	*p*-Value ^c^
Introduction of solids ^a^	<6 mo	Reference		Reference	
	≥6 mo	0.72 (0.23–2.51)	0.590	0.59 (0.12–2.74)	0.510
Disability status ^b^	Non-disability	Reference		Reference	
	Suspected	0.55 (0.20–1.42)	0.220	0.56 (0.19–1.55)	0.280
	Referred	0.37 (0.11–1.05)	0.080	0.35 (0.10–1.07)	0.080
	Confirmed	0.18 (0.05–0.52)	0.003 *	0.17 (0.04–0.60)	0.010 *
Preterm birth ^b^	Full-term	Reference		Reference	
	Preterm	0.22 (0.06–0.61)	0.010 *	0.28 (0.08–0.79)	0.030 *
Hispanic ethnicity ^b^	No	Reference		Reference	
	Yes	4.93 (1.91–15.32)	0.002 *	3.88 (1.28–13.59)	0.020 *
Birth weight (pounds) ^b^		1.47 (1.17–1.92)	0.002 *	1.39 (1.08–1.85)	0.020 *

^a^ The multivariable model was adjusted for disability status, ethnicity (Hispanic or Non-Hispanic), pre-term birth, and birth weight in pounds. ^b^ The multivariable model was adjusted for English proficiency and the country of birth. ^c^ Any *p*-value under 0.05 is considered statistically significant. * Statistically significant.

## Data Availability

Data are unavailable to protect the privacy of the participants.

## References

[1] Center for Disease Control and Prevention (2022). Overweight & Obesity. cdc.gov. https://www.cdc.gov/obesity/childhood/index.html.

[2] Abbasi A., Juszczyk D., van Jaarsveld C.H.M., Gulliford M.C. (2017). Body Mass Index and Incident Type 1 and Type 2 Diabetes in Children and Young Adults: A Retrospective Cohort Study. J. Endocr. Soc..

[3] Ward Z.J., Long M.W., Resch S.C., Giles C.M., Cradock A.L., Gortmaker S.L. (2017). Simulation of Growth Trajectories of Childhood Obesity into Adulthood. N. Engl. J. Med..

[4] American Academy of Pediatrics (2021). Infant Food and Feeding. aap.org. https://www.aap.org/en/patient-care/healthy-active-living-for-families/infant-food-and-feeding/.

[5] Huh S.Y., Rifas-Shiman S.L., Taveras E.M., Oken E., Gillman M.W. (2011). Timing of solid food introduction and risk of obesity in preschool-aged children. Pediatrics.

[6] Yuan M., Lu M., Guo Y., Lam K.B.H., Lu J., He J., Shen S., Wei D., Thomas G.N., Cheng K.K. (2022). Timing of Infant Formula Introduction in Relation to Body Mass Index and Overweight at Ages 1 And 3 Years: The Born in Guangzhou Cohort Study (BIGCS). Br. J. Nutr..

[7] Barrera C.M., Perrine C.G., Li R., Scanlon K.S. (2016). Age at Introduction to Solid Foods and Child Obesity at 6 Years. Child. Obes..

[8] Pearce J., Taylor M.A., Langley-Evans S.C. (2013). Timing of the introduction of complementary feeding and risk of childhood obesity: A systematic review. Int. J. Obes..

[9] Isong I.A., Rao S.R., Bind M.A., Avendaño M., Kawachi I., Richmond T.K. (2018). Racial and Ethnic Disparities in Early Childhood Obesity. Pediatrics.

[10] Aghaee S., Deardorff J., Quesenberry C.P., Greenspan L.C., Kushi L.H., Kubo A. (2022). Associations between Childhood Obesity and Pubertal Timing Stratified by Sex and Race/Ethnicity. Am. J. Epidemiol..

[11] Ogden C.L., Fryar C.D., Hales C.M., Carroll M.D., Aoki Y., Freedman D.S. (2018). Differences in Obesity Prevalence by Demographics and Urbanization in US Children and Adolescents, 2013–2016. JAMA.

[12] Pineros-Leano M., Grafft N., Aguayo L. (2022). Childhood obesity risk factors by race and ethnicity. Obesity.

[13] Gnawali A. (2021). Prematurity and the Risk of Development of Childhood Obesity: Piecing Together the Pathophysiological Puzzle. A Literature Review. Cureus.

[14] Ou-Yang M.C., Sun Y., Liebowitz M., Chen C.C., Fang M.L., Dai W., Chuang T.W., Chen J.L. (2020). Accelerated weight gain, prematurity, and the risk of childhood obesity: A meta-analysis and systematic review. PLoS ONE.

[15] Bjørge T., Engeland A., Tverdal A., Smith G.D. (2008). Body mass index in adolescence in relation to cause-specific mortality: A follow-up of 230,000 Norwegian adolescents. Am. J. Epidemiol..

[16] Young N.E. (2021). Childhood Disability in the United States: 2019. 2021. Census.gov. https://www.census.gov/library/publications/2021/acs/acsbr-006.html.

[17] Walker M., Nixon S., Haines J., McPherson A.C. (2019). Examining risk factors for overweight and obesity in children with disabilities: A commentary on Bronfenbrenner’s ecological systems framework. Dev. Neurorehabilit..

[18] Fryar C.D., Carroll M.D., Gu Q., Afful J., Ogden C.L. (2021). Anthropometric Reference Data for Children and Adults: United States, 2015–2018.

[19] World Health Organization (2009). Child Growth Standards. https://www.who.int/toolkits/child-growth-standards/standards.

[20] Kuczmarski R.J., Ogden C.L., Guo S.S., Grummer-Strawn L.M., Flegal K.M., Mei Z., Wei R., Curtin L.R., Roche A.F., Johnson C.L. (2022). 2000 CDC Growth Charts for the United States: Methods and Development.

[21] Hales C.M., Freedman D.S., Akinbami L., Wei R., Ogden C.L. (2022). Evaluation of Alternative Body Mass Index (BMI) Metrics to Monitor Weight Status in Children and Adolescents with Extremely High BMI Using CDC BMI-for-Age Growth Charts.

[22] R Core Team (2022). R: A Language and Environment for Statistical Computing.

[23] Moorcroft K.E., Marshall J.L., McCormick F.M. (2011). Association between timing of introducing solid foods and obesity in infancy and childhood: A systematic review. Matern. Child Nutr..

[24] Vehapoglu A., Yazıcı M., Demir A.D., Turkmen S., Nursoy M., Ozkaya E. (2014). Early infant feeding practice and childhood obesity: The relation of breast-feeding and timing of solid food introduction with childhood obesity. J. Pediatr. Endocrinol. Metab. JPEM.

[25] Uwaezuoke S.N., Eneh C.I., Ndu I.K. (2017). Relationship Between Exclusive Breastfeeding and Lower Risk of Childhood Obesity: A Narrative Review of Published Evidence. Clin. Med. Insights Pediatr..

[26] Tambalis K.D., Mourtakos S., Panagiotakos D.B., Sidossis L.S. (2018). Association of Exclusive Breastfeeding with Risk of Obesity in Childhood and Early Adulthood. Breastfeed. Med. Off. J. Acad. Breastfeed. Med..

[27] Chiang K.V., Hamner H.C., Li R., Perrine C.G. (2020). Timing of Introduction of Complementary Foods—United States, 2016–2018. MMWR. Morb. Mortal. Wkly. Rep..

[28] Fuzzell L.N., LaJoie A.S., Smith K.T., Philpott S.E., Jones K.M., Politi M.C. (2018). Parents’ adherence to pediatric health and safety guidelines: Importance of patient-provider relationships. Patient Educ. Couns..

[29] Bandini L.G., Curtin C., Hamad C., Tybor D.J., Must A. (2005). Prevalence of overweight in children with developmental disorders in the continuous national health and nutrition examination survey (NHANES) 1999–2002. J. Pediatr..

[30] Ells L.J., Lang R., Shield J.P., Wilkinson J.R., Lidstone J.S., Coulton S., Summerbell C.D. (2006). Obesity and disability—A short review. Obes. Rev. Off. J. Int. Assoc. Study Obes..

[31] De S., Small J., Baur L.A. (2008). Overweight and obesity among children with developmental disabilities. J. Intellect. Dev. Disabil..

[32] Van Cleave J., Heisler M., Devries J.M., Joiner T.A., Davis M.M. (2007). Discussion of illness during well-child care visits with parents of children with and without special health care needs. Arch. Pediatr. Adolesc. Med..

[33] Pandey M., Maina R.G., Amoyaw J., Li Y., Kamrul R., Michaels C.R., Maroof R. (2021). Impacts of English language proficiency on healthcare access, use, and outcomes among immigrants: A qualitative study. BMC Health Serv. Res..

[34] Al Shamsi H., Almutairi A.G., Al Mashrafi S., Al Kalbani T. (2020). Implications of Language Barriers for Healthcare: A Systematic Review. Oman Med. J..

[35] Kibakaya E.C., Oyeku S.O. (2022). Cultural Humility: A Critical Step in Achieving Health Equity. Pediatrics.

[36] Martín-Calvo N., Goni L., Tur J.A., Martínez J.A. (2022). Low birth weight and small for gestational age are associated with complications of childhood and adolescence obesity: Systematic review and meta-analysis. Obes. Rev. Off. J. Int. Assoc. Study Obes..

[37] Vasylyeva T.L., Barche A., Chennasamudram S.P., Sheehan C., Singh R., Okogbo M.E. (2013). Obesity in prematurely born children and adolescents: Follow up in pediatric clinic. Nutr. J..

[38] Pringle K.G., Lee Y.Q., Weatherall L., Keogh L., Diehm C., Roberts C.T., Eades S., Brown A., Smith R., Lumbers E.R. (2019). Influence of maternal adiposity, preterm birth and birth weight centiles on early childhood obesity in an Indigenous Australian pregnancy-through-to-early-childhood cohort study. J. Dev. Orig. Health Dis..

[39] Hildebrand J.S., Ferguson P.L., Sciscione A.C., Grobman W.A., Newman R.B., Tita A.T., Wapner R.J., Nageotte M.P., Palomares K., Skupski D.W. (2022). Breastfeeding Associations with Childhood Obesity and Body Composition: Findings from a Racially Diverse Maternal-Child Cohort. Child. Obes..

